# EPS deficiency aggravates intestinal mucosal inflammation and dysbiosis through disrupting macrophage polarization balance in ulcerative colitis

**DOI:** 10.3389/fimmu.2026.1878025

**Published:** 2026-09-15

**Authors:** Fengqin Zhu, Guiyuan Jin, Lihao Shi, Yufen Qin, Honglu Xue, Fengxian Dai, Xueying Chen, Guangxi Zhou

**Affiliations:** 1 Department of Gastroenterology, Affiliated Hospital of Jining Medical University, Jining Medical University, Jining, Shandong, China; 2 Medical Research Center, Affiliated Hospital of Jining Medical University, Jining Medical University, Jining, Shandong, China; 3 Institute of Immunology and Molecular Medicine, Jining Medical University, Jining, Shandong, China; 4 Department of Cardiology, Affiliated Hospital of Jining Medical University, Jining Medical University, Jining, Shandong, China

**Keywords:** IBD - inflammatory bowel disease, inflammation, lincRNA-EPS, macrophage, ulcerative colitis

## Abstract

**Background:**

Ulcerative colitis (UC) is a chronic, immune-mediated inflammatory bowel disorder, and macrophages are essential for maintaining intestinal mucosal balance during UC. Long intergenic non-coding RNA–erythroid prosurvival (lincRNA-EPS) is a novel lincRNA identified as a key inflammatory regulator. Nonetheless, the specific role of EPS in the pathogenesis of UC remains unclear.

**Methods:**

To investigate the contribution of EPS to intestinal inflammation, a model of colitis induced by dextran sulfate sodium (DSS) was created using both EPS gene knockout (EPS^-/-^) mice and wild-type (WT) mice. The impact of EPS on macrophage activities was assessed through various methods including qRT-PCR, flow cytometry, and immunofluorescence. Biopsies of the colon were collected from individuals diagnosed with UC for the purpose of EPS detection.

**Results:**

EPS^-/-^ mice exhibited more severe intestinal mucosal inflammation after DSS treatment, which was characterized by an increase in inflammatory cell infiltration, suppressed recovery of tight junction proteins and microbiota balance. EPS inhibits M1 macrophage polarization while promoting M2 macrophage polarization both *in vitro* and *in vivo*. The expression of EPS was found to be decreased in the inflamed mucosa and peripheral blood mononuclear cell (PBMC) of individuals suffering from UC, showing a negative correlation with the level of disease activity.

**Conclusions:**

This study revealed a novel function of EPS in macrophages in regulating the M1/M2 balance, inflammation, and gut microbiota during the pathological process of UC, and targeting EPS may represent a promising therapeutic approach for UC.

## Introduction

Ulcerative colitis (UC) is defined as a chronic, immune-mediated inflammatory bowel disorder that is marked by widespread mucosal inflammation limited to the colon and rectum. Clinically, it usually manifests with symptoms such as bloody diarrhea, urgency, tenesmus, and abdominal discomfort, exhibiting a relapsing-remitting pattern (1). Prolonged inflammation associated with UC heightens the risk of developing colorectal cancer (2). Therefore, prompt and effective treatment is essential to avert irreversible harm and enhance the outcomes of patients with UC.

In colon specimens from both UC patients and relevant animal models, a significant presence of macrophages has been observed, which are crucial in initiating and resolving inflammation (3, 4). M1 macrophages are activated by interferon gamma (IFN-γ), bacterial lipopolysaccharide (LPS), or tumor necrosis factor alpha (TNF-α) to generate an array of pro-inflammatory cytokines, including interleukin-6 (IL-6). In contrast, M2 macrophages are stimulated by IL-4 and IL-13 to produce arginase (Arg)-1, Ym-1, and anti-inflammatory cytokines like IL-10. While M1 macrophages and pro-inflammatory cytokines exacerbate UC, M2 macrophages facilitate tissue repair and help resolve inflammation, thus alleviating UC symptoms (5–7). Given that dysfunctional polarization of macrophages is observed in the progression of UC, there is growing recognition of the therapeutic promise in targeting the main regulators of macrophage polarization to redirect their activity in the context of UC.

Past efforts have elucidated the roles of Long noncoding RNAs (lncRNAs) involved in the regulation of M1/M2 polarization. lncRNA AK083884 promotes macrophage M2 polarization and protects mice from CVB3-induced Viral myocarditis (8). lncRNA RP11-417E7.1 promotes colorectal cancer metastasis by activating the Wnt/β-catenin pathway and facilitating exosome-mediated M2 macrophage polarization (9). LncRNA MIR4435-2HG suppression inhibits macrophage M1 polarization while promoting M2 polarization, thereby alleviating intestinal inflammation in UC through JAK1/STAT1 signaling (10). However, the mechanism underlying the regulation of the M1/M2 switch and the development of UC is still incomplete and far from being systematic.

Long intergenic RNA–erythroid prosurvival (lincRNA-EPS), also known as Ttc39aos1, is a novel lncRNA identified as a key inflammatory regulator. EPS was reported to inhibit the expression of immune response genes as well as activation of NLRP3 in macrophages, mitigate the inflammatory level of vital organs, and decrease lethality after endotoxin challenge (11). EPS represses the HMGB1-NF-κB-dependent inflammation of pancreatic macrophages and exerts a protective effect on acute pancreatitis (12). EPS inhibits caspase-11 and NLRP3 inflammasomes in gingival fibroblasts to alleviate periodontal inflammation (13). EPS alleviates inflammation in temporomandibular joint osteoarthritis by binding to SRSF3 (14). However, whether and how EPS in macrophages regulates M1/M2 polarization, inflammation, and UC has remained elusive.

In this study, we firstly demonstrated that EPS is significantly downregulated in the intestinal mucosa and peripheral blood mononuclear cell (PBMC) of patients with UC and negatively correlated with disease activity. EPS gene knockout (EPS^-/-^) mice aggravated dextran sulfate sodium (DSS)-induced colitis, including more inflammatory cell infiltration, increased disruption of intestinal tight junction and microbiota balance. Moreover, EPS inhibits M1 macrophage polarization while promoting M2 macrophage polarization. Thus, we propose a function of EPS in macrophages in regulating the M1/M2 balance, inflammation, and gut microbiota during the pathological process of UC, and targeting EPS may represent a promising therapeutic approach for UC.

## Materials and methods

### DSS-induced colitis model in mice

EPS^-/-^ mice were generated by Viewsolid Biotechnology Co., Ltd. in Beijing, China. All animal experiments were reviewed and granted approval by the Institutional Animal Care and Use Committee at Jining Medical University. A colitis model was established as previously described (15). For each animal experiment, we utilized 8 to 10 mice per group. Briefly, groups consisting of wild-type C57BL/6 mice and EPS^-/-^ mice were administered with 2.0% DSS (MP Biomedicals, Solon, Ohio, USA) in their drinking water for a week. During this time, we monitored the symptoms of colitis in mice from each group, such as diarrhea, bloody stools, variations in body weight, and survival rates. On day eight, all mice were euthanized, colonic tissues and feces were collected for follow-up experiments.

### Mouse imaging

Mice underwent a fasting duration of 12 hours before dissection and were given an oral dose of FITC-dextran (50 mg/kg). After 4 hours, a small-animal imaging system (PerkinElmer IVIS Spectrum, USA) was utilized to evaluate the distribution of FITC-dextran. Peripheral blood samples were harvested by retro-orbital sinus puncture from the mice and the supernatant serum were obtained after centrifuge. Then, 100 μL of the above serum was added to a 96-well plate, and the serum’s fluorescence intensity was measured by a fluorescence microplate reader.

### Sequencing analysis of gut microbiota

Gut microbiota analysis was performed by Shanghai Ouyi Biotechnology Co., LTD. (Shanghai, China). In brief, genomic DNA was isolated from the fecal samples of the above mice using the MagPure Soil DNA LQ Kit. 16S rRNA gene of the bacterial was amplified using polymerase chain reaction (PCR). Subsequently, the PCR product was purified with AMPure XP beads and measured using Qubit to adjust suitable sequencing concentration. Sequencing analysis of the microbiota was executed on the Illumina NovaSeq 6000 platform.

### Isolation and culture of macrophages

Peritoneal macrophages (PMs) and bone marrow macrophages (BMDMs) were isolated from EPS^-/-^ mice and WT mice as previously described (15). In brief, peritoneal exudate cells were collected by performing lavage of the peritoneal cavity using Dulbecco’s Modified Eagle Medium (DMEM). These cells were incubated in DMEM medium enriched with 10% FBS and 1% penicillin/streptomycin for 3 hours in order to gain PMs. To drive BMDMs, bone marrow cells were extracted from the femurs and tibias of WT and EPS^-/-^ mice and were cultured in a medium containing granulocyte-macrophage colony-stimulating factor (10 ng/mL) for 7 days. These macrophages were then plated into 12-well plates for following experiments.

### Quantitative reverse transcription PCR

Total RNA extraction was performed utilizing Trizol reagent (Invitrogen). Reverse transcription to cDNA was implemented with the aid of a 5x All-In-One RT MasterMix kit (abm). Then, the quantitative reverse transcription PCR (qRT-PCR) were performed using a fluorescent qPCR kit (Vazyme). mRNA expression were assessed using the SYBR Green and 2^-ΔΔCt^ methodologies.

### Detection of macrophage polarization

PMs and BMDMs obtained from WT and EPS^-/-^ mice were treated with different stimulations to promote M1-type or M2-type macrophage polarization. LPS (200 ng/mL) and interferon (IFN)-γ (20 ng/mL) were applied for 72 hours to promote M1-type macrophage polarization, and IL-4 (20 ng/mL) and IL-13 (20 ng/mL) were applied for 72 hours to encourage M2-type macrophage polarization. For polarization detection, we use flow cytometry and qRT-PCR to test specific markers expression.

### Patients

Participants diagnosed with UC and healthy control subjects (HCs) were recruited from the Department of Gastroenterology at the Affiliated Hospital of Jining Medical University in Jining, Shandong, China, during the period from May 2022 to May 2025. Colonic tissues and peripheral blood samples were collected. We employed recognized metrics, such as the Mayo score, the ulcerative colitis endoscopic severity index (UCEIS), and the Dublin index (16) to evaluate the disease’s severity of UC. In addition, we collected some clinical parameters associated with UC, such as C-reactive protein (CRP), hemoglobin (Hb), platelet (Plt) counts, neutrophil and lymphocyte ratio, et al, to assess their correlation with EPS expression.

### Statistical analysis

Statistical evaluations were performed utilizing GraphPad Prism version 8. Each experiment was carried out with a minimum of three replicates. The analysis of data involved the unpaired Student’s t-test or one-way ANOVA followed by the Tukey Multiple Comparison Test. The relationships between EPS expression and clinical indicators relevant to UC was assessed using Spearman correlation. A *p*-value of less than 0.05 was considered statistically significant.

## Results

### EPS deficiency aggravates DSS-induced colitis in mice

To explore the role of EPS in colonic inflammation, we developed a mouse model of colitis induced by DSS, which included both WT and EPS^-/-^ mice. As shown in **Figures 1A, B**, the survival rate of EPS^-/-^ mice was markedly lower than that of their WT counterparts over an 8-day observation period, accompanied by significantly greater body weight loss. On the eighth day, all subjects were euthanized, and it was found that the colons of EPS^-/-^ mice were notably shorter in length compared to those of WT mice (see **Figures 1C, D**). Additionally, histological evaluations revealed that EPS^-/-^ mice presented higher pathological scores relative to WT mice after DSS treatment (**Figures 1E, F**).

**Figure 1:**
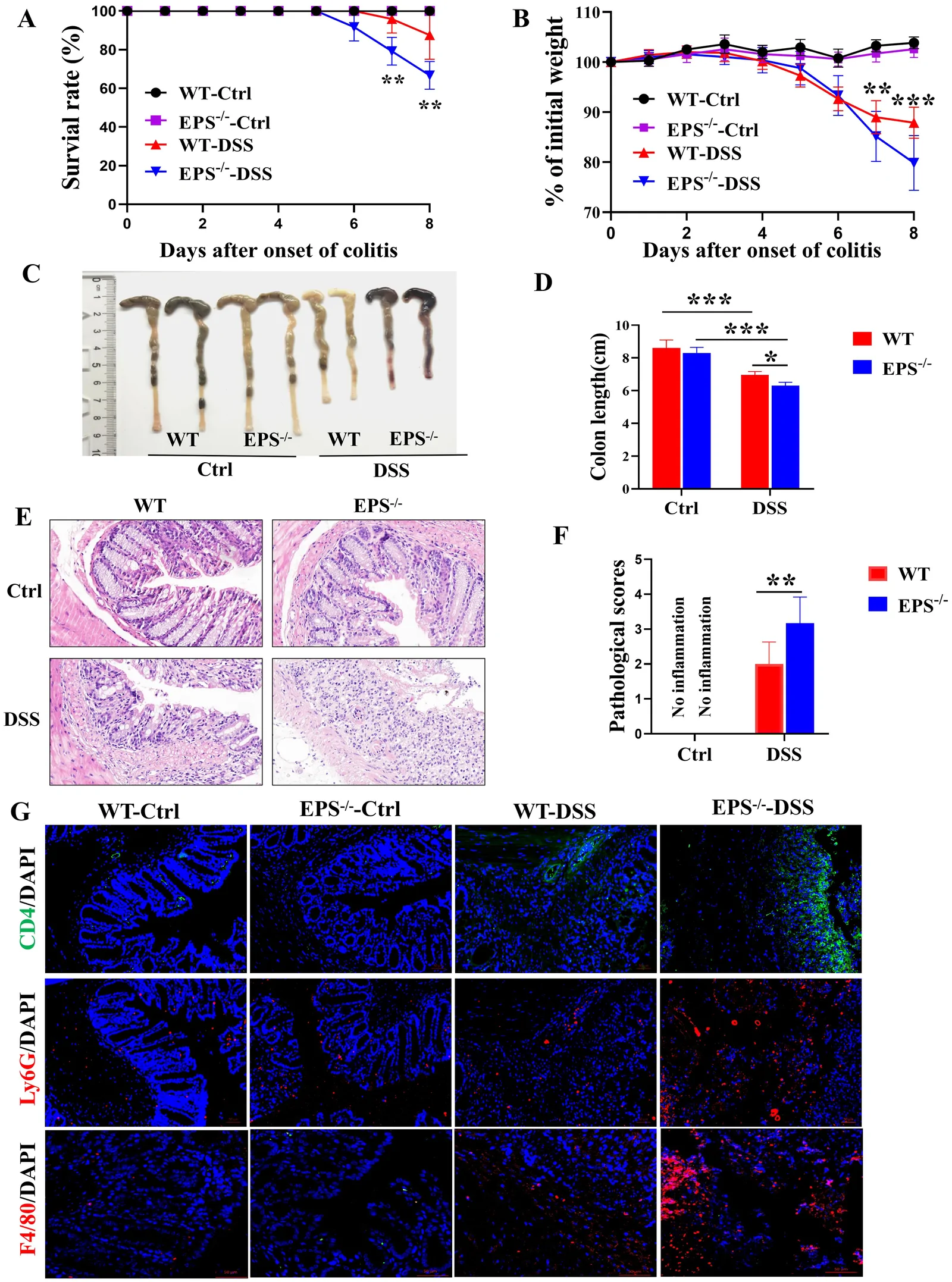
EPS deficiency aggravates dextran sulfate sodium (DSS)-induced colitis in mice. WT and EPS^-/-^ mice (n = 8–10 in each group) were given 2% DSS in drinking water for continuous 7 days and then followed with regular water for another 1 day. On Day 8, all mice were sacrificed. **(A)** The survival rate in both groups after DSS insult. **p < 0.01. **(B)** The changes in body weight were expressed as a percentage of the original weight at the start of the experiment during a period of 8-day observation. **p < 0.01, and ***p < 0.001. **(C)** Gross morphology of the colons when mice were sacrificed. **(D)** The statistical length of colons in different groups. *p < 0.05, and ***p < 0.001. **(E)** Representative H&E staining images of distal colonic sections (original magnification × 200). **(F)** The changes of pathological scores from colonic sections were calculated as indicated. **p < 0.01. **(G)** Cellular fractions of CD4^+^ T cells, Ly6G^+^ neutrophils, and F4/80^+^ macrophages in colonic mucosa were determined by immunohistochemistry staining. Scale bars = 50 μm.

To enhance the understanding of the degree of intestinal inflammation, we investigated the infiltration of immune cells in the colonic lamina propria and evaluated the integrity of the intestinal barrier using immunofluorescent staining techniques. Our findings revealed that the levels of CD4^+^ T cells, Ly6G^+^ neutrophils, and F4/80^+^ macrophages were increased in the DSS-treated groups compared to the control groups. Additionally, when comparing the WT DSS group to the EPS^-/-^ DSS mice, we noted heightened levels of CD4^+^ T cells, Ly6G^+^ neutrophils, and F4/80^+^ macrophages in EPS^-/-^ DSS group (**Figure 1G**). These findings indicated that EPS exhibited significant anti-inflammatory therapeutic effectiveness *in vivo*.

### EPS deficiency exacerbates intestinal mucosal barrier damage in colitis mice

Disruption of the intestinal barrier is a critical aspect of UC; therefore, we examined how EPS affect the intestinal barrier’s functionality. We firstly assessed the expression of tight junction proteins ZO-1, Occludin, and E-cadherin in the intestinal mucosal tissue via immunofluorescence. Our findings revealed a greater degree of destruction in these proteins within the EPS^-/--^DSS group compared to the WT-DSS group (**Figure 2A**). Subsequently, we detected the permeability of the intestinal mucosal barrier. On day 8, all experimental mouse groups were administered FITC-dextran, a tracer used to assess permeability, via oral route. 4 hours later, we evaluated the distribution of FITC-dextran within the abdominal cavity and measured its serum concentration. As illustrated in **Figure 2B**, the DSS groups exhibited a pronounced fluorescent signal in the abdominal cavity, indicating significant damage to the intestinal mucosal barrier. Moreover, the EPS^-/--^DSS group displayed an even greater fluorescence intensity compared to the WT-DSS group, suggesting that a lack of EPS exacerbates intestinal barrier impairment during colitis. Additionally, the serum concentration of FITC-dextran was found to be highest in EPS^-/-^ colitis mice (**Figure 2C**), aligning with the abdominal fluorescence intensity results.

**Figure 2:**
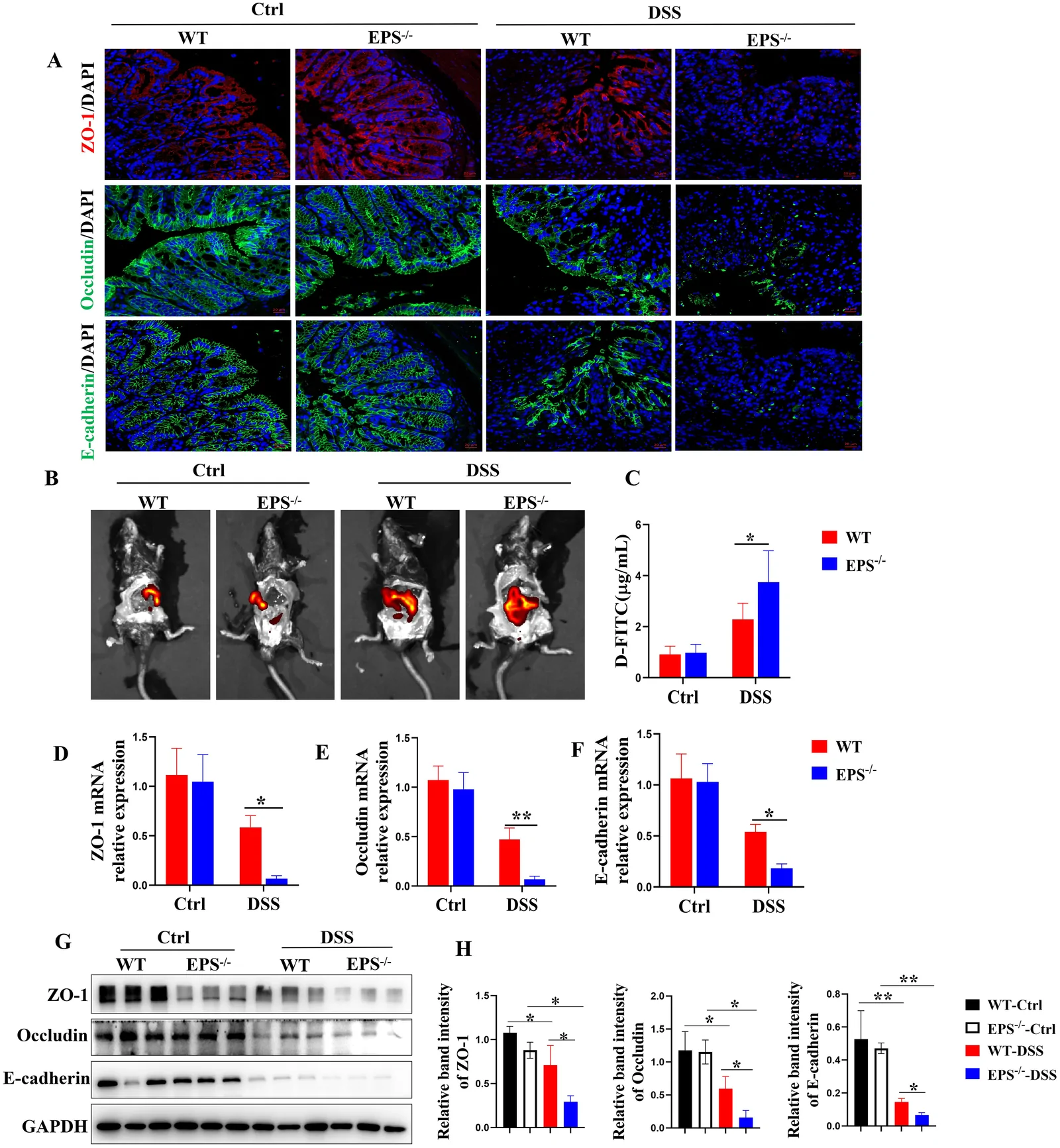
EPS deficiency destroy the intestinal mucosal barrier in mice with DSS-induced colitis. A colitis model was established in WT and EPS^-/-^ mice using 2.0% DSS. **(A)** Immunofluorescence detection of tight junction proteins ZO-1, Occludin, and E-cadherin in intestinal mucosal tissues. Scale bars = 20 μm. **(B)** FITC-dextran distribution in mice observed using a small-animal imaging system. **(C)** Serum FITC-dextran levels measured using a microplate system. **(D–F)** Expression of tight junction proteins ZO-1 **(D)**, Occludin **(E)**, and E-cadherin **(F)** in primary intestinal epithelial cells from colitis mice detected using qRT-PCR. *p < 0.05, **p < 0.01. **(G)** Expression of tight junction proteins ZO-1, Occludin, and E-cadherin in primary intestinal epithelial cells detected using Western blotting. **(H)** Western blot band intensities in G were quantified using Image J software and normalized to GAPDH. *p < 0.05, and **p < 0.01.

Furthermore, we extracted intestinal epithelial cells from the mucosal tissues of the aforementioned experimental mice to conduct additional tests on the mRNA and protein expressions of tight junction proteins. The data indicated a significant reduction in the levels of ZO-1, Occludin, and E-cadherin in the intestinal epithelial cells derived from EPS^-/--^DSS mice relative to those from WT-DSS mice (**Figures 2D–H**), thereby confirming that the absence of EPS worsened the damage to the intestinal mucosal barrier in colitis-affected mice. These *in vivo* findings illustrated that EPS plays a beneficial role in maintaining intestinal barrier function during colitis.

### EPS deficiency worsens intestinal microbiota dysbiosis in colitis mice

Dysbiosis in the intestinal microbiota plays a significant role in the development of UC (17). To investigate whether EPS can improve dysbiosis of the intestinal microbiota, 16S rRNA sequencing was performed to evaluate the diversity and composition of the gut microbiota. α-diversity metrics reflect the richness and abundance of microbial communities. In terms of α-diversity, the DSS group displayed reduced levels in the PD-whole-tree, Chao1, Shannon, and ACE indices, indicating a decline in the diversity of microbial community distribution when compared to the control groups. Additionally, a decrease in the PD-whole-tree, Chao1, Shannon, and ACE indices was observed in the EPS^-/--^DSS group when contrasted with the WT-DSS group, underscoring the significant impact of EPS on the microbiome’s richness (**Figure 3A**). In addition, Non-metric multi-dimensional scaling (NMDS), a β-diversity index, exhibited impairment in the EPS^-/--^DSS group relative to the WT-DSS group, highlighting the crucial influence of EPS on the richness of the microbiome (**Figure 3B**).

**Figure 3:**
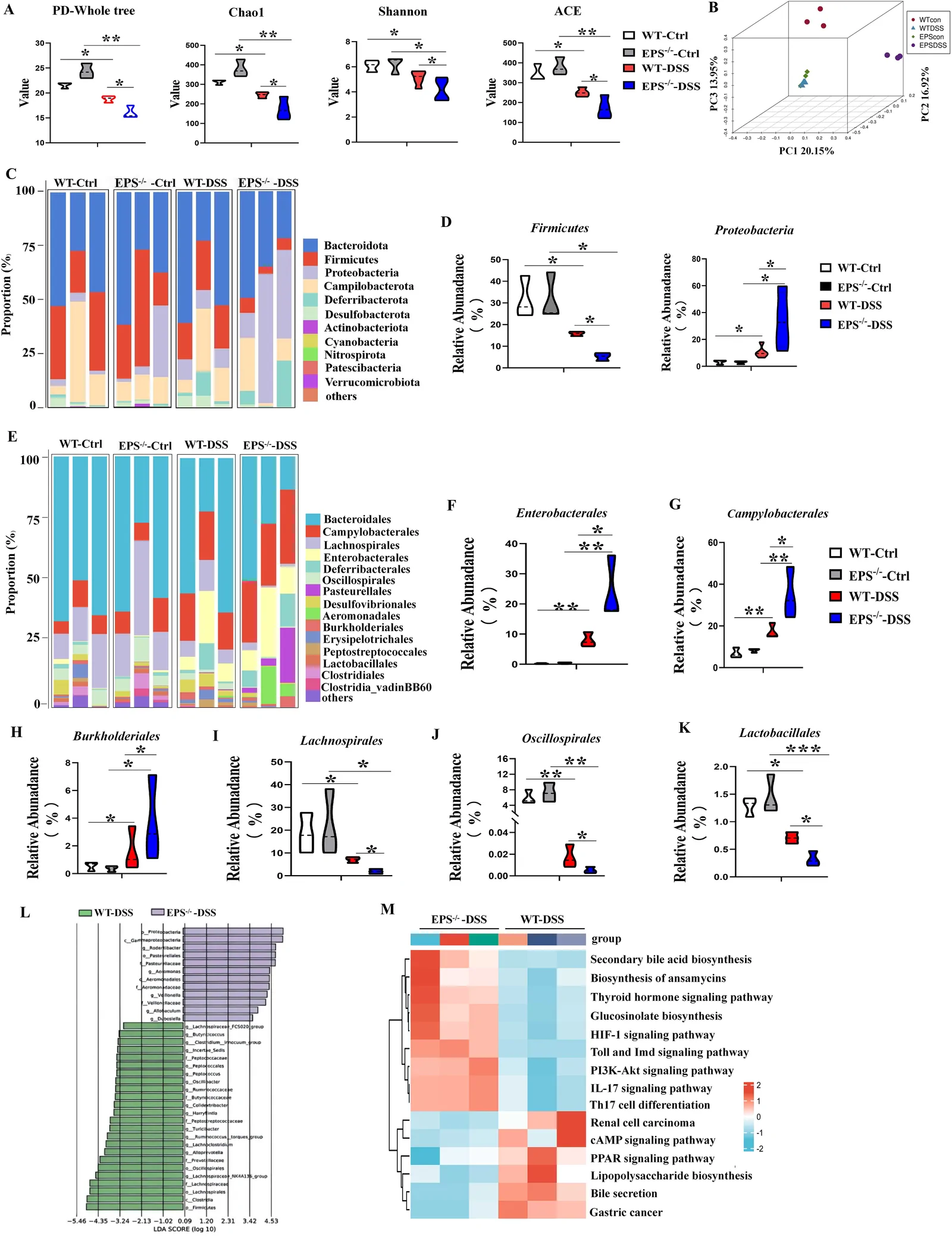
EPS deficiency sharpens intestinal microbiota dysbiosis in DSS-induced colitis. WT and EPS^-/-^ mice (n = 8–10 in each group) were given 2.0% DSS to establish a colitis model, and the feces of all mice were collected for 16S rRNA sequencing. **(A)** The α-diversity of the microbiome among all groups depicted according to PD-whole-tree, Chao1, Shannon and ACE index. *p < 0.05, **p < 0.01. **(B)** The β-diversity index of the microbiome among all groups showing as Non-metric multi-dimensional scaling (NMDS). **(C)** Phylum-level gut microbiota profile. **(D)**The relative abundance of *Proteobacteria* and *Firmicutes*. *p < 0.05. **(E)** Order-level gut microbiota profile. **(F–K)** The relative abundance of *Enterobacterales, Campylobacterales, Burkholderiales*, *Lachnospirales, Oscillospirales*, and *Lactobacillales.* *p < 0.05, **p < 0.01, and ***p < 0.001. **(L)** Linear discriminant analysis effect size (LEfSe) results. **(M)** KEGG annotation and functional enrichment of the divergent microbiota.

We also investigated the composition and prevalence of bacteria at various hierarchical levels. At the phylum tier, the gut microbiota in both the control and DSS mice predominantly comprised *Bacteroidota*, *Firmicutes*, and *Proteobacteria*. A notable rise in *Proteobacteria* was seen in the DSS group when compared to the control, while there was a reduction in *Firmicutes*. Interestingly, within the EPS^-/--^DSS group, the levels of *Proteobacteria* increased, and *Firmicutes* decreased relative to the WT-DSS group (**Figures 3C, D**). Furthermore, the abundance of bacteria at the order level is illustrated in **Figure 3E**. In the EPS^-/--^DSS group, the relative abundance of *Enterobacterales*, *Campylobacterales*, and *Burkholderiales*-all under the *Proteobacteria* phylum, showed the most significant increase, while *Lachnospirales, Oscillospirales*, and *Lactobacillales*-falling under the *Firmicutes* phylum, experienced the most substantial decrease (**Figures 3F–K**). The results of the linear discriminant analysis effect size were consistent with those obtained from the analysis of variance (**Figure 3L**). Furthermore, by utilizing KEGG annotation and functional enrichment, we discovered that these altered microbiota were associated with several inflammation-related signaling pathways, including HIF-1, PI3K-Akt, Toll and Imd, IL-17, cAMP, and PPAR (**Figure 3M**). Interestingly, we noticed that PI3K-Akt, cAMP, and PPAR signaling pathways were associated to macrophages differentiation. In summary, the knockdown of EPS enhances the dysbiosis in mice with DSS-induced colitis, indicating that EPS aids in the restoration of a healthier microbial community and may be linked to macrophages immune response.

### EPS expression is suppressed in macrophages exposed to LPS and promotes the expression of pro-inflammatory cytokines in macrophages

Macrophages play a crucial role in maintaining intestinal homeostasis and seem to act as drivers of inflammation, in the context of UC (18). Previous studies that myeloid cells including macrophages and dendritic cells express a high level of EPS (11, 19), and our study found that the changes in the bacterial community caused by EPS may be linked to macrophages immune response. Thus, we speculated that EPS regulates intestinal mucosal inflammation and microbiota disruption might through modulating macrophage immune responses in UC. To this end, we began by isolating PMs from WT mice, followed by stimulation with LPS for 3 hours, and subsequently analyzed their transcriptome. Notably, several established inflammatory genes, such as IL-6, IL-1β, CCL22, and TNF, were significantly upregulated following LPS stimulation. Interestingly, a number of lncRNAs exhibited an expression pattern that was completely contrary to those inflammatory genes, showing suppression after LPS treatment. Among these, EPS, previously identified as a transcriptional brake that mitigates inflammation, was one of the key lncRNAs that demonstrated an inverse correlation with the aforementioned inflammatory gene (**Figure 4A**). Additionally, we observed that the expression of EPS was significantly decreased in both LPS-stimulated PMs and BMDMs over a time gradient (**Figures 4B, C**). But whether EPS could affect macrophage immune responses is still not clear.

**Figure 4:**
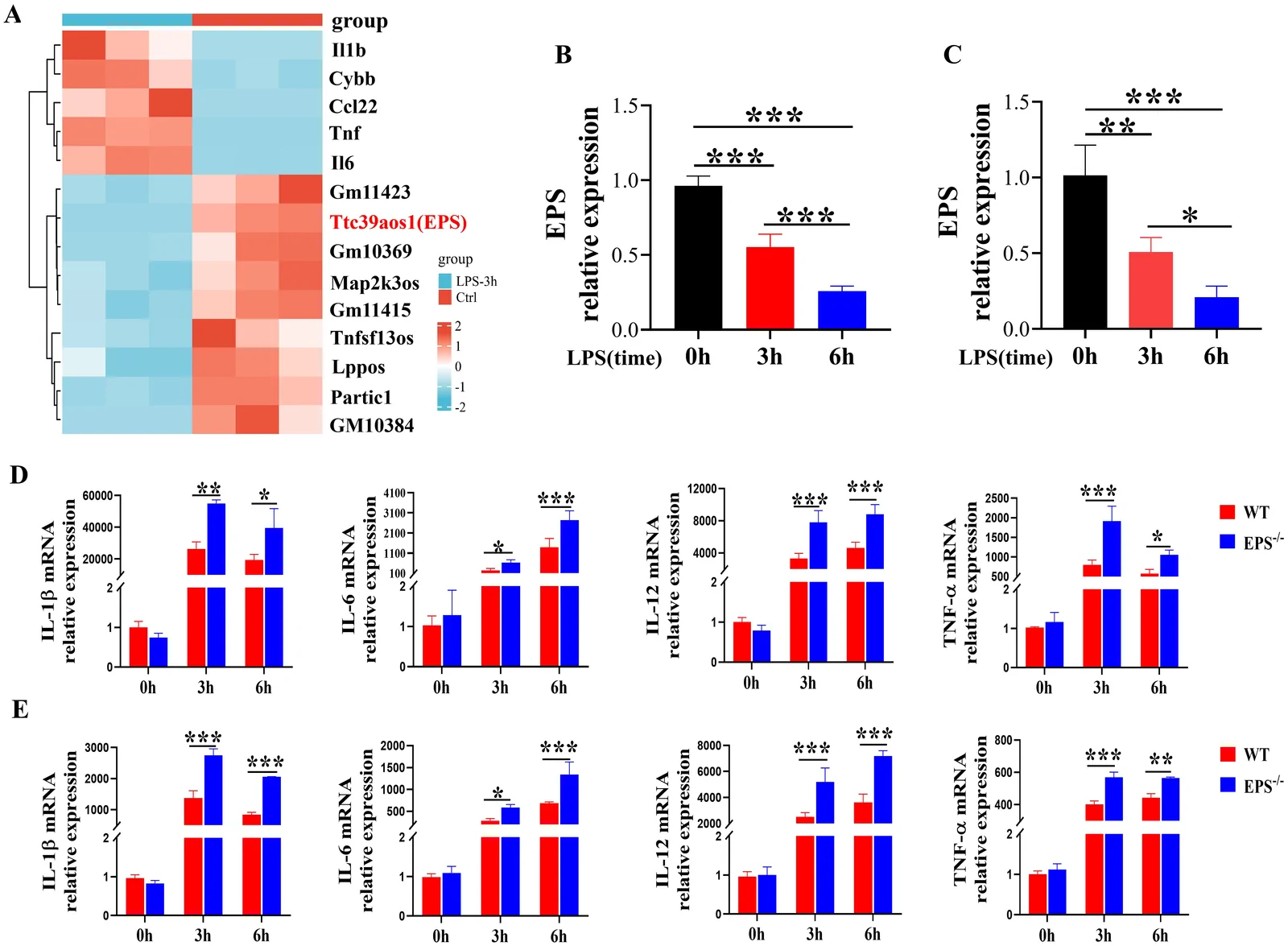
EPS expression is downregulated in macrophage under LPS-stimulated inflammatory conditions and suppresses the expression of pro-inflammatory genes in macrophage. **(A)** PMs were isolated from WT mice and stimulated *in vitro* by LPS (500 ng/mL) for 0, and 3 hours. PMs were collected, and total RNA was extracted for RNA-Seq analysis. Heatmap of the representative inflammatory genes and lncRNAs. **(B, C)** EPS expression in PMs **(B)** and BMDMs **(C)** after LPS stimulation for 0, 3 and 6 hours. *p < 0.05, **p < 0.01, ***p < 0.001. **(D, E)** PMs **(D)** and BMDMs **(E)** were isolated from WT and EPS^-/-^ mice and stimulated *in vitro* by LPS (500 ng/mL) for 0, 3, and 6 hours. Cells were collected, and total RNA was extracted. The mRNA levels of inflammatory-related genes IL-1β, IL-6, IL-12, and TNF-α were detected by qRT-PCR. *p < 0.05, **p < 0.01, ***p < 0.001. Experiments were performed in at least triplicate for each group.

A variety of researches have indicated that heightened levels of pro-inflammatory cytokines, including IL-1β, IL-6, and TNF-α, in macrophages following inflammatory triggers play an essential role in the development of intestinal inflammation (19). To explore how EPS influence the expression of inflammatory cytokines in macrophages during inflammation, we isolated PMs and BMDMs, stimulating them with LPS at intervals of 0, 3, and 6 hours. We subsequently measured the mRNA levels of different inflammatory cytokines using qRT-PCR. The results revealed that a deficiency in EPS considerably heightened the levels of pro-inflammatory cytokines (IL-1β, IL-6, IL-12, and TNF-α) in both PMs and BMDMs when compared to the control group (**Figures 4D, E**). These findings suggest that EPS deficiency promotes LPS-induced pro-inflammatory response in macrophages.

### EPS deficiency promotes M1 macrophage polarization *in vitro* and in the colon of colitis mice

Taking into account the atypical polarization of macrophages that occurs during the progression of UC, we explore the role of EPS in the polarization of macrophages. Initially, we characterized M1 macrophages *in vivo* within the colonic tissues of mice with DSS-induced colitis. Immunofluorescence staining revealed a rise in F4/80^+^ CD86^+^ M1 macrophages in the colons of EPS knockout mice subjected to DSS treatment (**Figure 5A**). Next, the mRNA levels of M1-associated genes, including iNOS, IL-1β, and TNF-α were significantly enhanced in the intestinal mucosal tissue from EPS^-/--^DSS mice (**Figure 5B**).

**Figure 5:**
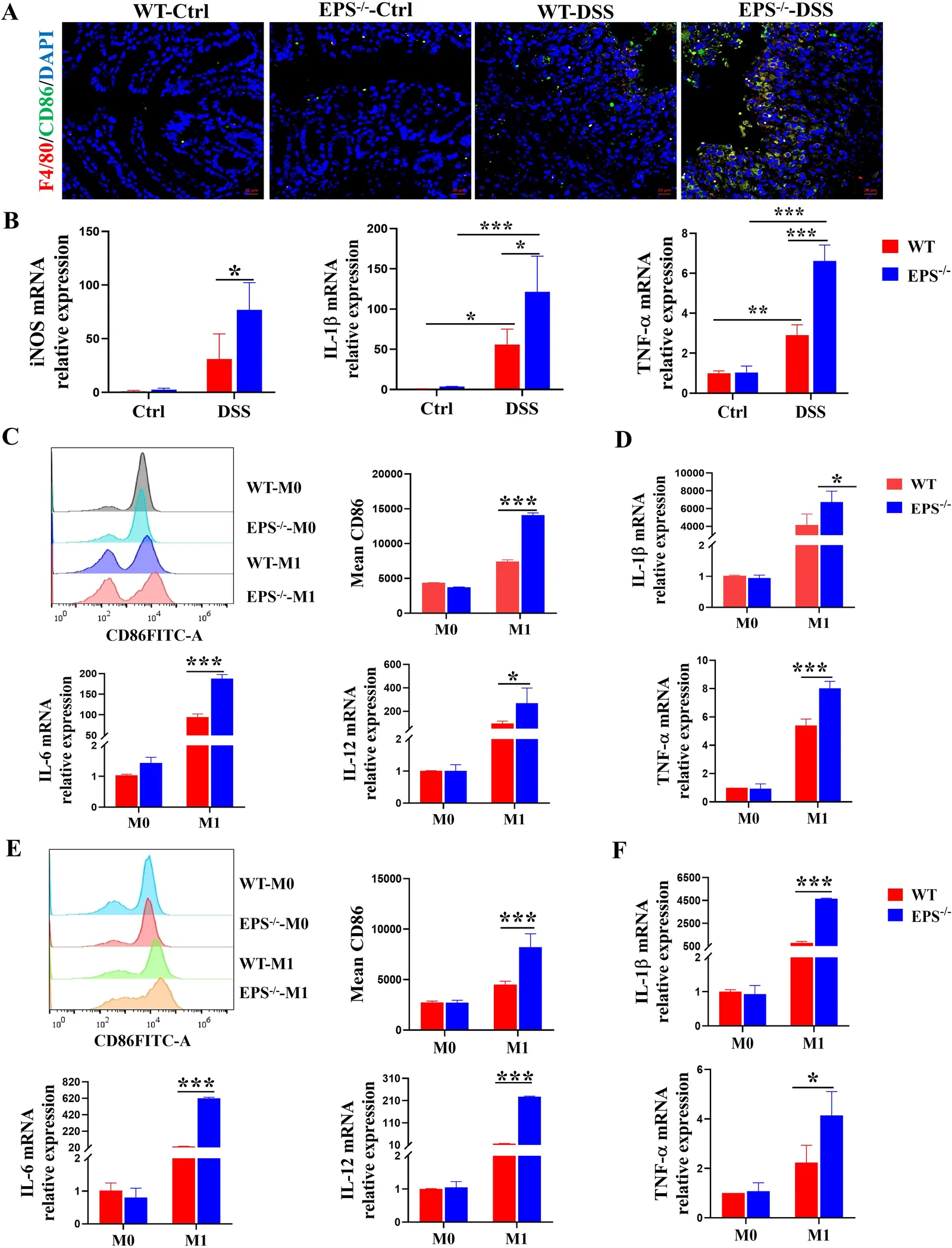
EPS deficiency enhances M1 macrophage polarization *in vitro* and in the Colon of UC. **(A)** Immunostaining analysis of CD86^+^ F4/80^+^ macrophages in colonic tissues from DSS-induced colitis mice. Scale bars = 20 μm. **(B)** The mRNA levels of iNOS, IL-1β, and IL-12 in immune cells from colonic lamina propria were detected by qRT-PCR. *p < 0.05, **p < 0.01, ***p < 0.001. **(C–F)** PMs and BMDMs were isolated from WT and EPS^-/-^ mice, and induced to polarize into M1 type macrophages. **(C)** Mean fluorescence intensity of CD86 in PMs was detected by flow cytometry. ***p < 0.001. **(D)** Gene expression of some M1 markers: IL-1β, IL-6, IL-12, and TNF-α in PMs was detected by qRT-PCR. *p < 0.05, ***p < 0.001. **(E)** Mean fluorescence intensity of CD86 in BMDMs by flow cytometry. ***p < 0.001. **(F)** Gene expression of IL-1β, IL-6, IL-12, and TNF-α in BMDMs was detected by qRT-PCR. *p < 0.05, ***p < 0.001. Experiments were performed in at least triplicate for each group.

To further validate the role of EPS in promoting the induction of M1 macrophages, we treated macrophages *in vitro* with LPS and IFN-γ. Our observations revealed a notable rise in the mean fluorescence intensity of CD86, as measured by flow cytometry in PMs lacking EPS, suggesting enhanced polarization towards the M1 macrophage phenotype (**Figure 5C**). Aligned with our *in vivo* findings, qRT-PCR assessment revealed an increased expression of M1-type macrophage markers such as IL-1β, IL-6, IL-12, and TNF-α in PMs deficient in EPS (**Figure 5D**). The findings suggest that insufficient EPS encourages macrophages to polarize towards the M1 phenotype. Similar results were observed in BMDMs, in which the fluorescence intensity of CD86 was increased, and the expression of M1-type markers was elevated in EPS^-/-^ BMDMs compared to the control group (**Figures 5E, F**).

### EPS deficiency weakens M2 macrophage polarization *in vitro* and in the colon of colitis mice

M2 macrophages facilitate tissue healing and the resolution of inflammation, which alleviates symptoms of UC (20). Our *in vivo* findings indicated that the lack of EPS led to a reduction in the population of F4/80^+^ CD206^+^ M2 macrophages within the intestinal mucosal tissue (**Figure 6A**), as well as a decrease in the mRNA expression of M2-related genes such as Ym1, Arg1, and CD206 in the colons of DSS-treated mice (**Figure 6B**).

**Figure 6:**
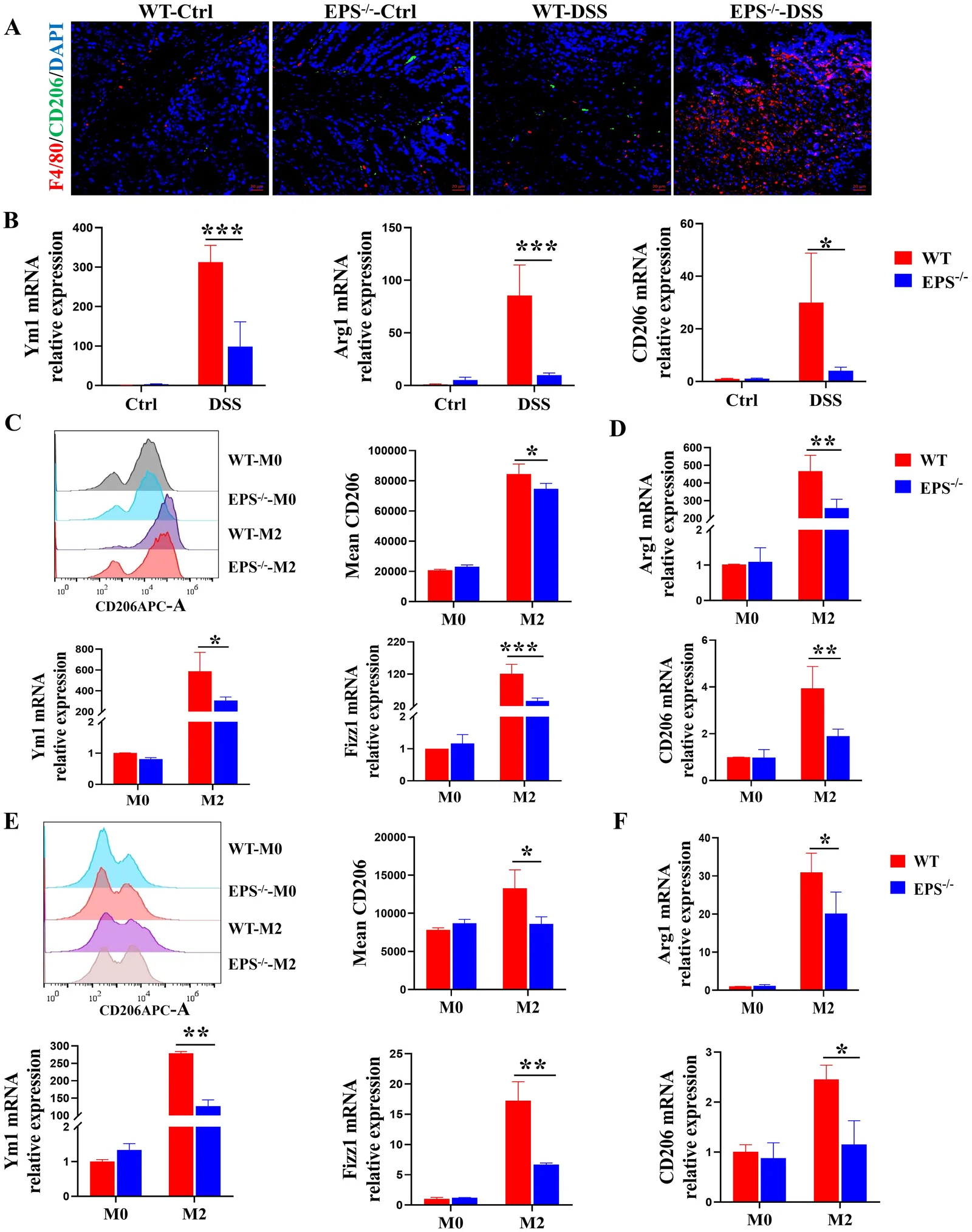
EPS deficiency restrains M2 macrophage polarization *in vitro* and in the Colon of UC. **(A)** Immunostaining analysis of CD206^+^ F4/80^+^ macrophages in colonic tissues from DSS-induced colitis mice. Scale bars = 20 μm. **(B)** The mRNA levels of Ym1, Arg1, and CD206 in immune cells from colonic lamina propria were detected by qRT-PCR. *p < 0.05, and ***p < 0.001. **(C–F)** PMs and BMDMs were isolated from WT and EPS^-/-^mice, and induced to polarize into M2 type macrophages. **(C)** Mean fluorescence intensity of CD206 in PMs was detected by flow cytometry. *p < 0.05. **(D)** Gene expression of Arg1, Ym1, Fizz1, and CD206 in PMs was detected by qRT-PCR. *p < 0.05, **p < 0.01, and ***p < 0.001. **(E)** Mean fluorescence intensity of CD206 in BMDMs by flow cytometry. *p < 0.05, and **p < 0.01. **(F)** Gene expression of Arg1, Ym1, Fizz1, and CD206 in BMDMs was detected by qRT-PCR. *p < 0.05, and **p < 0.01. Experiments were performed in at least triplicate for each group.

Consequently, we examined whether EPS facilitates M2 polarization in PMs and BMDMs. Following induction by IL-4 and IL-13, PMs lacking EPS showed a notable reduction in the average fluorescence intensity of CD206 (**Figure 6C**). A deficiency in EPS also led to decreased mRNA levels of M2-specific markers, including Arg1, Ym1, Fizz1, and CD206 (**Figure 6C**). These findings indicate that the absence of EPS effectively hinders M2-type macrophage polarization. Consistent with these observations, the expression of M2-related genes was diminished in EPS^-/--^BMDMs (**Figures 6E, F**).

### EPS is downregulated in inflamed colonic mucosa and PBMCs of patients with active UC

To explore the role of EPS in the progression of UC, we analyzed the expression of EPS in colonic biopsy specimens and PBMCs from individuals with active UC. The results from qRT-PCR indicated a notable reduction in EPS levels within the intestinal mucosa and PBMCs of patients suffering from active UC in comparison to HCs (**Figures 7A, B**). Additionally, we investigated the correlation between EPS expression in the intestinal mucosa and a range of clinical parameters associated with UC, such as CRP, Hb, Plt, MES, the Mayo score, UCEIS, and Dublin score, among others. As illustrated in **Figures 7C, D**, a negative correlation was identified between EPS expression and clinical measurements including stool blood score, UCEIS, CRP, MES, Mayo score, and Dublin score in patients diagnosed with UC, indicating a potential negative relationship between EPS expression and UC disease intensity. Overall, these results suggest that EPS plays a role in the pathophysiological mechanisms of UC and might serve as a promising therapeutic target for this condition.

**Figure 7:**
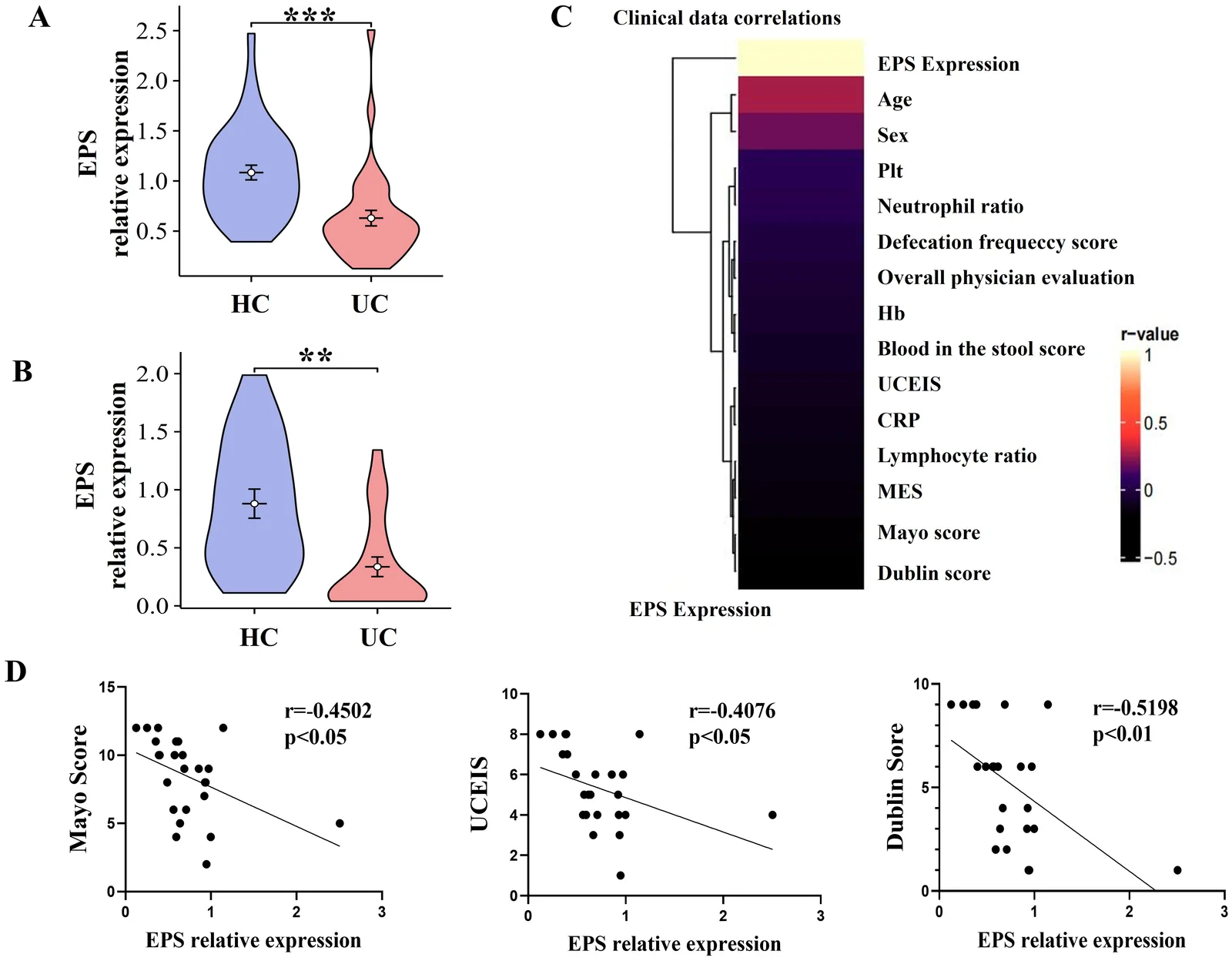
Expression of EPS is downregulated in inflamed colon mucosal biopsies and PBMCs of UC patients. **(A)** The levels of EPS in the inflamed mucosa of patients with active UC and HCs were assessed using qRT-PCR. RNA was extracted from colon biopsy samples collected from HC individuals (n = 37), and those with active UC (n = 35). Gene expression was normalized to GAPDH in each group. ***p < 0.001. **(B)** Expression of EPS in PBMCs of HCs (n = 25), and patients with UC (n = 28) was detected by qRT-PCR. **p < 0.01. **(C)** Spearman’s correlation coefficient values were computed to analyze the relationship between EPS expression in the intestinal mucosal tissues of UC patients and various clinical indicators. **(D)** Correlation analysis between the Mayo Score, UCEIS, and Dublin Score and EPS expression in the inflamed mucosa of patients with UC.

## Discussion

UC represents one of the two main types of inflammatory bowel disease. Worldwide, it impacts around 5 million individuals; however, the underlying causes and mechanisms of UC are still not well understood, and there is currently no definitive cure (21). Current UC treatments largely rely on aminosalicylates, corticosteroids, immunomodulators, and biological therapies, which are frequently accompanied by drug resistance, systemic immunosuppression, and recurrent inflammation (22–25). lncRNAs are garnering increased interest for their roles in regulating immunological and inflammatory processes (26–28). lincRNA-EPS serves as a crucial regulator in the context of inflammation (11, 29). We initially observed that EPS expression was reduced in the inflamed colonic mucosa and PBMCs of patients experiencing UC. EPS deficiency promoted M1 polarization of macrophages while limited M2 polarization, thus aggravated intestinal tissue damage and inflammatory infiltration. These findings indicate that EPS-mediated immune regulation is indispensable for maintaining intestinal mucosal tolerance. Therefore, EPS may serve as a potential novel therapeutic target and auxiliary biomarker for UC.

Dysregulation of the balance of M1 and M2 macrophages is a hallmark of UC (30). Adjusting macrophage polarization could serve as a potent approach for managing UC. Multiple lncRNAs have been reported to orchestrate intestinal macrophage polarization during gut inflammation. For instance, lncRNA Gm16023 limits M1 macrophage-mediated hyperglycemia-exacerbated septic intestinal injury via miR-377-3p/Sirt1 axis (31). LncRNA MIR4435-2HG suppression regulates macrophage M1/M2 polarization and reduces intestinal inflammation in mice with ulcerative colitis (10). LncRNA NEAT1 facilitates M1 polarization of intestinal macrophages (32). To the best of our knowledge, the current study is the first to investigate and discover that lincRNA-EPS regulates intestinal macrophage polarization. We indicate that EPS mitigates the progression of colitis by modulating the equilibrium between pro-inflammatory M1 and anti-inflammatory M2 macrophages. Our present research offers evidence that EPS inhibits colitis via a macrophage-dependent mechanism, primarily by modulating the balance between M1 and M2 macrophages *in vivo*. To validate our *in vivo* observations regarding the effects of EPS on macrophage polarization, PMs and BMDMs from EPS knockout and wild-type mice were subjected to LPS & IFN-γ or IL-4 & IL-13 treatments *in vitro* to directly investigate the macrophage polarization process. The findings revealed that EPS significantly reduced the polarization of M1 macrophages while also enhancing M2 macrophages polarization in mouse PMs and BMDMs, suggesting that EPS plays a role in reestablishing the balance between M1 and M2 macrophages. However, the direct molecular binding target of EPS on macrophages remains uncharacterized in the current work. Further biochemical assays such as pull-down and SPR are required to screen and validate potential receptor candidates, which will be addressed in our subsequent investigations.

Intestinal macrophages, gut microbiota and the intestinal mucosal barrier engage in intricate bidirectional crosstalk to maintain intestinal homeostasis, and collectively play a central role in the initiation and resolution of UC. Intestinal macrophages are now increasingly recognized to regulate epithelial homeostasis, maintain the mucus barrier, and interact with the gut microbiota (33, 34). In the context of UC, aberrant activation of macrophages disrupts mucus barrier integrity (35). Aberrantly activated macrophages secrete proinflammatory cytokines that diminish mucin secretion and disrupt the mucus barrier, rendering the intestinal epithelium more susceptible to injury and microbial invasion (36). Additionally, intestinal macrophages may influence the composition of the gut microbiota, which in turn can impact mucus layer integrity (37, 38). Conversely, gut microbiota can modulate macrophage activity, thereby shaping intestinal barrier function. Aeromonas residing in the gut and their secreted aerolysin contribute to the depletion of intestinal macrophages, disruption of the intestinal epithelial barrier, and ultimately drive UC (39). We found that EPS restores the balance between M1 and M2 macrophages, mitigates intestinal barrier injury and microbiota disruption. However, the precise closed-loop mechanism remains to be further elucidated in future investigations. In the setting of colitis, intestinal epithelial injury and cell sloughing lead to the loss of epithelial cells, which may also decrease the mRNA abundance of epithelial-derived genes. Thus, we cannot dissect whether EPS acts directly on IECs or exerts its protective effect exclusively via macrophage-intrinsic responses. Future work including IEC-specific and macrophage-specific EPS knockout models, will be required to formally distinguish epithelial-cell-autonomous versus macrophage-mediated functions of EPS. Further, our 16S rRNA sequencing demonstrated more pronounced DSS-triggered gut microbial perturbations in EPS^-/-^ mice compared with WT controls, but we cannot determine whether the shifts in gut microbiota contribute to aggravated colitis, or are merely a secondary outcome of severe intestinal injury. Fecal microbiota transplantation experiments will be needed to verify potential causality.

Notably, our current study cannot establish definite causal relationships between EPS, macrophages and colitis progression. We adopted global EPS knockout mice rather than macrophage-specific conditional knockout strains, and functional experiments using freshly isolated primary intestinal macrophages are absent. Therefore, our results mainly provide correlative evidence. Further studies incorporating primary macrophage culture and conditional knockout mouse models are required to verify the cell-autonomous function of EPS in macrophages during intestinal inflammation. In addition, we adopted LPS stimulation to establish the inflammatory macrophage model in our *in vitro* cellular experiments. Nevertheless, UC is regarded as a sterile non-infectious intestinal disease predominantly driven by DAMPs including ATP and HMGB1, instead of microbial-originated LPS. Therefore, the LPS-treated *in vitro* system cannot fully recapitulate the sterile inflammatory microenvironment of UC. Further validation with DAMP stimuli will be required to confirm our key findings, which will be pursued in future investigations.

In conclusion, our study emphasizes the critical role of EPS on intestinal mucosal inflammation by regulating the inflammatory response of macrophages in UC. EPS mitigates intestinal inflammation and microbiota disruption by suppressing the expression of pro-inflammatory genes, and restoring the balance between M1 and M2 macrophages. Thus, we demonstrates that EPS may be an effective therapeutic approach to treat intestinal inflammation of UC. Future translational studies focusing on EPS-targeted intervention, including lncRNA stabilization, targeted delivery, and macrophage-specific activation strategies, may provide new avenues for precise and safe UC treatment.

## Data Availability

The original contributions presented in the study are publicly available. This data can be found in the NCBI BioProject database under accession number PRJNA1526041.
